# Nursing Outside the Lines: Identity, Policy, and the Rise of Gig Economy Nursing

**DOI:** 10.1155/jonm/4925153

**Published:** 2026-03-18

**Authors:** Animesh Ghimire

**Affiliations:** ^1^ School of Nursing and Midwifery, Faculty of Medicine, Nursing and Health Science, Monash University, Wellington Rd, Clayton, Victoria, 3800, Australia, monash.ac.za; ^2^ Sustainable Prosperity Initiative Nepal, Baneshwor-31, Kathmandu, Nepal; ^3^ School of Nursing, and School of Public Health, Chitwan Medical College, Bharatpur-5 Kailashnagar, Chitwan, Nepal, cmc.edu.np

**Keywords:** agency nursing, Australia, casualization, gig economy nursing, health policy, professional identity, workforce sustainability

## Abstract

**Background:**

Australia’s nursing workforce is under intensifying strain, yet an increasing subset of registered nurses (RNs) are not seeking permanence—they are choosing to work exclusively through agencies. This trend is commonly framed as a matter of flexibility or pay. However, such explanations understate what exclusive agency nursing does to professional identity, how it operates as a response to organizational dysfunction, and how policy–practice arrangements enable transient careers within a regulated high‐income system.

**Objectives:**

To generate an interpretive, policy‐relevant account of how exclusive casual agency employment shapes nurses’ professional identities, motivations, career trajectories, and engagement with collective professional advocacy.

**Methods:**

An interpretivist, constructionist qualitative design was employed. Semistructured interviews were conducted with 16 Melbourne‐based RNs who worked exclusively through nursing agencies. Data were analyzed using reflexive thematic analysis (RTA), and the manuscript was reported in line with the Reflexive Thematic Analysis Reporting Guidelines (RTARG).

**Results:**

Five themes were constructed. Exclusive agency nursing involved the crafting of a professional self “beyond the ward,” marked by transactional workplace relationships, strategic emotional boundary‐setting, and a shift toward adaptability as a core competence. Agency work was frequently narrated as strategic withdrawal and resistance to permanent‐role conditions—bureaucratic overload, inflexible rostering, and unsupportive leadership cultures associated with burnout. Participants situated their careers within a “casualization ecosystem” in which organizational reliance on contingent labor, agency intermediation, and policy/funding dynamics collectively sustain demand for agency nurses. Yet, this work model generated persistent ambivalence about long‐term sustainability and was perceived to fragment professional belonging and weaken collective voice in advocacy and industrial representation.

**Conclusions:**

Exclusive agency nursing is not simply an individual preference for flexibility; it is an identity project and a system‐level signal of deteriorating employment conditions in permanent roles. Workforce responses that focus only on recruitment incentives or short‐term staffing solutions risk entrenching casualization and its downstream effects.

**Implications for Nursing Management:**

Nurse leaders should treat agency reliance as both a staffing strategy and an organizational diagnostic. Reforming permanent roles requires actionable changes—credible roster flexibility, visible supportive leadership, and working conditions that reduce burnout—alongside structured integration practices for agency nurses to protect team cohesion, care continuity, and professional sustainability.

## 1. Introduction

The nursing workforce constitutes an essential component of healthcare systems globally and is especially crucial in high‐income countries (HICs) facing escalating demands from aging populations, the rising prevalence of chronic conditions, maintaining regulatory laws such as the optimum nurse–patient ratio, and the impacts of global health crises such as the COVID‐19 pandemic [1, 2]. These pressures strain health service capacity and place immense demands on nurses—the clinicians delivering care under often challenging circumstances. Consequently, ensuring a stable, skilled, and sustainable nursing workforce is paramount to maintaining accessible, high‐quality healthcare. However, achieving this goal is becoming increasingly complex amid current global challenges [3, 4].

Australia provides a compelling case study of these dynamics. Its healthcare system, featuring the publicly funded universal scheme “Medicare” alongside a private sector, depends heavily on a large, highly trained healthcare workforce [5, 6]. Data from the Australian Institute of Health and Welfare (AIHW) highlight that within public hospitals alone, approximately 448,000 full‐time equivalent (FTE) staff were employed in 2022–23. Nurses form the largest professional group in these settings, representing 42% (186,000 FTE) of this workforce, compared to 13% (56,700 FTE) for salaried medical officers [7]. Internationally, Australia maintains one of the highest nurse‐to‐population ratios, recorded at approximately 13.1 FTE nurses and midwives per 1000 people in 2022 [8].

However, this high national ratio masks structural and demographic pressures that help explain why shortages persist. Registrant data indicate marked geographic maldistribution: Most nurses and midwives practice in metropolitan areas with far fewer in remote communities, and the average age rises with remoteness—amplifying replacement pressures outside major cities [9]. The register also skews toward mid‐career and older cohorts (e.g., sizeable proportions aged 55–64 and 65–74), meaning retirements and reductions in working hours intersect with rising service demand from population aging and increasing care complexity [10]. Reflecting this, national modeling informing Australia’s *Draft National Nursing Workforce Strategy* projects that even with continued growth in both supply and demand, supply will not keep pace—yielding an undersupply of around 70,700 FTE nurses by 2035 (demand rising from 334,873 FTE in 2023 to 493,282 in 2035; supply from 324,989 to 422,575 FTE) [11]. The domestic education pipeline has remained relatively stable (with 19,817 commencing domestic nursing enrollments in 2023); however, recent new‐graduate applications have softened, increasing reliance on internationally educated nurses as a crucial supplement (e.g., 16,155 of 38,854 first‐time nurse registrants in 2024/25) [10, 12]. Recognizing these constraints, recent Commonwealth reforms have sought to reduce regulatory delays and expedite registration pathways for internationally qualified nurses.

Against this background of systemic strain and projected shortages, fundamental shifts in nurses’ employment patterns are evident. The traditional model, characterized by permanent positions within a single organization [13, 14], faces challenges from the rise of nontraditional work arrangements [15, 16], including workforce casualization and increased reliance on agency nursing [17]. While Australia does not routinely publish national participation rates for nurses working “agency‐only,” jurisdictional indicators signal escalating reliance on agency/casual labor: in the state of Victoria, public hospital spending on overtime and agency nurses/midwives more than double from under AUD 100 million in 2018 to over AUD 211 million in 2022 [18, 19]; in NSW Health, inquiry‐related workforce reporting documented an average of 481.6 agency‐nurse FTE engaged across the system in 2023 (with higher utilization in rural/remote districts) and sustained high agency expenditure beyond pandemic peaks [20, 21]. This shift is frequently discussed within the broader context of the “gig economy” impacting healthcare internationally, including other HICs such as the United States and the United Kingdom, as well as middle‐income settings such as India and China [22–25]. In Australia, this trend accelerated significantly following the COVID‐19 pandemic, which intensified burnout and prompted many nurses to prioritize greater control over schedules, improve work–life balance, and, in some cases, the higher hourly compensation offered by casual or agency roles [26].

Existing research has started to map this landscape, identifying key motivations that draw nurses to “agency” or “gig” work, such as flexibility, autonomy, diverse experiences, and financial incentives [27]. Simultaneously, studies highlight significant drawbacks, including income precarity, the absence of benefits such as paid leave and retirement contributions common in permanent roles, potential professional isolation, and concerns about care continuity [28, 29]. However, much of this foundational work, often relying on quantitative methods focused on motivation or satisfaction, provides limited insight into the qualitative reasoning and lived experiences of nurses making these choices.

Consequently, a critical gap persists in our understanding, necessitating a nuanced, qualitative exploration. While we know that nurses are choosing exclusive agency work, key questions remain regarding *how* they navigate this complex terrain. There is limited insight into how this work model shapes professional identity negotiation beyond surface‐level satisfaction metrics. Furthermore, how the specific Australian policy, funding, and organizational practice ecosystem facilitates or sustains these transient careers warrants exploration from the perspective of those embedded within it. Finally, the critical implications for nurses’ long‐term career sustainability and their engagement in professional advocacy and collective action remain poorly understood aspects of this workforce shift. Addressing this gap requires moving beyond quantifying motivations toward an interpretive understanding of the lived experiences and meaning‐making processes characterizing this significant segment of the nursing workforce.

This paper reports on a qualitative study designed to address this knowledge gap. Using reflexive thematic analysis (RTA) [30], this research explores the experiences, motivations, identities, career perspectives, and systemic perceptions of registered nurses who work exclusively through agencies in Melbourne, Australia. By centering the voices of these nurses, the study sought to generate rich interpretive insights into the complexities of navigating contemporary nursing careers “*outside the lines*” of traditional employment structures. Understanding these lived realities is crucial as the profession confronts profound internal shifts amidst demanding external pressures, challenging conventional notions of nursing work and identity. There is a pressing need to understand the multifaceted reality of casual agency nursing beyond its surface appearances, prompting us to consider the profound shifts reshaping the profession and the systemic forces at play.

## 2. Theoretical Orientation and Research Aim

### 2.1. Analytic Approach and Theoretical Framework

This study employs RTA, as conceptualized by Braun and Clarke [31], as the analytic methodology. Furthermore, the reporting of this research within this manuscript follows the principles detailed in the Reflexive Thematic Analysis Reporting Guidelines (RTARG) [30, 32] to promote methodological coherence and reflexive openness (Supporting File 1). RTA itself is grounded in a “Big Q” qualitative paradigm [33], which fundamentally diverges from postpositivist assumptions common in quantitative or more positivist‐oriented qualitative research. It moves away from seeking singular truths and from relying on measures such as intercoder reliability to validate findings [30]. Instead, RTA explicitly values the researcher’s subjectivity, viewing it not as a bias to be eliminated but as an essential analytic resource—the very tool through which interpretation and meaning‐making occur [30].

This approach is particularly well‐suited for the present study, which seeks to explore the complex, multifaceted, and often subtle lived experiences of registered nurses working exclusively via agencies in Melbourne, Australia. RTA offers the theoretical flexibility and analytical depth needed to investigate this phenomenon. The study is situated within an interpretivist and constructionist framework [34], viewing participants’ experiences and identities not as fixed realities but as socially constructed meanings, shaped by context and articulated through their accounts. Language is thus treated not as a transparent window onto reality, but as constitutive of it. Consistent with a contextualist, constructionist, RTA, I did not apply a single “unifying” theory as a deductive template; instead, I drew on complementary theoretical resources as sensitizing concepts to deepen the interpretation of participants’ accounts. Transaction Cost Economics (TCE) helps illuminate the “transactional” character of agency arrangements by foregrounding how organizations and workers may prefer contracting over internal employment under uncertainty and in response to coordination/monitoring costs, an approach increasingly used to interpret contingent staffing in healthcare [35, 36]. Social exchange theory and psychological contract scholarship provide a relational lens for understanding “opting out” as a response to perceived breakdowns in reciprocity and support (e.g., psychological contract breach), which is associated with nurses’ trust, satisfaction, and turnover intentions [35, 37, 38]. Meaningful‐work perspectives further inform interpretation of narratives in which nurses described reclaiming purpose by privileging clinical care and minimizing bureaucratic demands, aligning with meaning‐based explanations of withdrawal and turnover [39]. The analytic focus attends to both semantic content—the explicit descriptions of work experiences, choices, and challenges—and latent meanings, exploring underlying assumptions, values, identity negotiations, and subtle critiques embedded within participants’ narratives [33, 40]. The overall goal in employing RTA is to develop a rich, contextualized, and interpretive understanding of how these nurses navigate casual agency work, construct their professional identities, and perceive the enabling ecosystem within Melbourne’s healthcare system.

### 2.2. Researcher Positioning and Reflexive Practice

Central to the integrity of RTA is sustained researcher reflexivity [41]. As the sole author and researcher for this study, I inevitably shape the research process and interpretation. My dual role as a registered nurse and an academic gives me an insider and an analyst perspective. Furthermore, I have navigated roles in both permanent hospital settings and as a casual agency nurse, which enriches this viewpoint. This shared identity likely fosters enhanced rapport and a deeper, more nuanced understanding of participants’ experiences and the specific language they used [42]. It allowed me to probe effectively based on shared contextual knowledge.

At the same time, this insider positioning required sustained critical self‐awareness to ensure I engaged with participants’ accounts as situated meanings, rather than treating my own experience as the default interpretive frame [43]. I used reflexive journaling and analytic memoing throughout dataset generation and analysis to document my assumptions, emotional responses, and evolving interpretive decisions, and to actively interrogate how my standpoint might be shaping what I was noticing and how I was coding [31]. For example, I initially expected participants’ narratives to foreground flexibility and pay as primary drivers of exclusive agency work; however, repeated accounts of organizational unsupportiveness, burnout cultures, and perceived expendability in permanent roles required me to revisit early codes and refine developing themes so that “flexibility” was interpreted within a broader patterned meaning of strategic boundary setting and resistance to system failures. In line with RTARG guidance, I did not use participant validation/member checking as an “accuracy” test; instead, I used “member reflections” by sharing a brief thematic summary with participants and inviting reflections and elaborations [31], which I treated as additional interpretive material that deepened analytic insight rather than as verification. I also sought structured critique from a single “critical friend”—a nursing academic and registered nurse with approximately 30 years’ experience across diverse roles, including agency nursing—who did not independently code transcripts or provide consensus checks but engaged in challenging dialog about my coding decisions, theme boundaries, and plausible alternative readings [31, 44]. Collectively, these practices were not intended to achieve neutrality but to make my interpretive role visible and to use my subjectivity critically and transparently as an analytic resource [33].

### 2.3. Research Aim

This study was guided by the following research question: How do Registered Nurses working exclusively via agencies in Melbourne experience and understand their professional identity, motivations, career trajectories, and engagement with the broader profession and healthcare system?

## 3. Methodology

This section details the methodological approach employed in this study, outlining the setting, participant selection and characteristics, ethical considerations, procedures for dataset generation, and the process of data analysis using RTA.

### 3.1. Study Setting, Participant Selection, and Characteristics

#### 3.1.1. Study Setting

This research was situated within the urban healthcare environment of metropolitan Melbourne, Victoria, Australia, a major center in a HIC with a mixed public–private health system. Nursing work in this setting is shaped by national regulation and state‐based industrial and policy settings that govern employment arrangements, staffing expectations, and workforce mobility [45, 46]. Recruitment was conducted through two private‐sector nursing agencies with national operations (i.e., Australia‐wide agencies) that actively place nurses into services across metropolitan Melbourne. Both agencies function as generalist providers of casual nursing labor—facilitating placements across a range of typical agency settings (e.g., acute hospital wards, aged care, and community contexts) rather than operating as niche, specialty‐only agencies. Using nationally operating agencies—common intermediaries within Australia’s contemporary casual staffing market—provided a context well suited to explore how the interplay between personal career negotiation, organizational staffing practices, and the broader sociopolitical landscape of healthcare in Victoria shapes individual nurses’ experiences of agency‐only work.

#### 3.1.2. Participant Selection Rationale

Consistent with the interpretivist–constructionist orientation of this study and the “Big Q” commitments of RTA, participant selection was guided by the aim of developing a dataset with sufficient informational richness to support an in‐depth, contextualized interpretation of nurses’ experiences of working exclusively via agencies, rather than achieving statistical representativeness. The participant group size (*n* = 16) was therefore justified using qualitative sampling logic aligned with information power: The study focus was specific (agency‐only RN work), participants were information‐rich with direct experience of the phenomenon, interviews were detailed (60–90 minutes) and generated substantive accounts, and the analytic aim prioritized interpretive depth over breadth [47, 48]. Recruitment and analysis proceeded iteratively, and I judged the dataset sufficient when the developing thematic account stabilized—that is, no substantively new dimensions relevant to the study aims emerged within the dataset. Deviant/negative cases could be meaningfully integrated without requiring additional themes or categories. In line with guidance that data saturation is not conceptually coherent for RTA, I do not claim “saturation;” instead, I argue that this participant group provided adequate informational power and interpretive depth to address the research question and construct a nuanced thematic account [47].

#### 3.1.3. Recruitment and Selection Process

Participants were recruited through an open call using a digital flyer that included study information and a QR code linking to the plain‐language statement. The flyer was distributed via the social media platforms of two nationally operating nursing agencies that place nurses into services across metropolitan Melbourne. Because recruitment occurred through open advertising (rather than direct invitations), the total number of nurses who viewed the advertisement (i.e., the denominator of “potential respondents contacted”) could not be determined. No incentive or reimbursement was offered; participation was entirely voluntary and without reward.

Over the recruitment period, 21 nurses expressed interest by contacting the researcher via email. Following initial contact, I (AG) undertook a brief eligibility screening via email and/or phone using a structured checklist aligned with the inclusion criteria. This screening involved confirming: (a) current RN registration with the Australian Health Practitioner Regulation Agency (AHPRA), (b) current engagement in nursing work exclusively through one or more agencies, and (c) no concurrent permanent full‐time or part‐time nursing employment. Where relevant, I also clarified the nurse’s primary practice context(s) (e.g., acute care, aged care, and community) and their capacity to participate in a remote interview. Of those who expressed interest, three were ineligible (most commonly due to holding concurrent permanent employment), and two did not respond to follow‐up. Sixteen eligible nurses provided written informed consent electronically and completed an interview, and no participants withdrew.

#### 3.1.4. Reflexive Considerations in Recruitment

As the sole researcher and an RN with prior experience in both permanent and agency roles, specific strategies were employed to mitigate potential power dynamics during recruitment. The use of an open advertisement (flyer) and requiring participants to initiate contact ensured self‐selection and reduced potential coercion. All recruitment materials explicitly emphasized the voluntary nature of participation, confidentiality, and the right to withdraw without consequence. Despite these efforts, a notable challenge was the difficulty in recruiting male nurses, resulting in a final participant group of 3 men and 13 women. While reflecting broader nursing trends with male nurses comprising 12.1% of the nursing workforce and 87.9% identifying as female [49], this demographic skew potentially limits the insights into male perspectives on agency work.

#### 3.1.5. Participant Group Characteristics

The participant group comprised 16 registered nurses (3 male and 13 female), assigned pseudonyms (RN1–RN16) to protect anonymity. Participants’ nursing experience since initial registration ranged from approximately 3 to 30+ years, and their time working exclusively as casual agency nurses ranged from approximately 1 to 12 years. Participants reported working across a range of clinical settings typical for agency nurses in metropolitan Melbourne, including acute medical/surgical wards, emergency departments, critical care units, aged care facilities, and community health settings. Key participant characteristics are summarized in Table 1.

**TABLE 1:** Participants’ sociodemographic characteristics.

Participant	Age (years)	Gender	Previous education	Years of nursing (approx.)	Years working exclusively as agency nurse (approx.)	Primary area(s) of practice via agency
RN 1	28	Female	Bachelor of Nursing	6	2	Medical/Surgical, Emergency Dept (ED)
RN 2	39	Female	Bachelor of Nursing	12	4	Intensive Care Unit (ICU), Critical Care
RN 3	48	Female	Bachelor of Nursing	18	6	Aged Care, Community Nursing
RN 4	26	Male	Bachelor of Nursing	4	2	ED, Medical Ward
RN 5	31	Female	Master of Science Nursing (MSN)	8	3	Mental Health
RN 6	59	Female	Bachelor of Nursing	25	10	Operating Theatre, Post‐Anaesthesia Care Unit (PACU)
RN 7	29	Female	Bachelor of Nursing	7	2	Surgical Ward, Medical Ward
RN 8	38	Male	Bachelor of Nursing	15	5	ICU, ED
RN 9	62	Female	Master of Science Nursing (MSN)	30+	12	Critical Care (ICU/CCU)
RN 10	38	Female	Bachelor of Nursing	10	4	Community Nursing, Aged Care
RN 11	27	Female	Bachelor of Nursing	5	2	Medical/Surgical, Rehabilitation
RN 12	53	Female	Bachelor of Nursing	22	8	Aged Care
RN 13	30	Male	Bachelor of Nursing	7	2	Mental Health, ED
RN 14	39	Female	Master of Science Nursing (MSN)	16	5	Palliative Care, Oncology
RN 15	25	Female	Bachelor of Nursing	2	1	Surgical Ward
RN 16	48	Female	Master of Science Nursing (MSN)	14	8	Paediatrics, Special Care Nursery

### 3.2. Ethical Considerations

This study was conducted in accordance with the ethical principles outlined in the Declaration of Helsinki. Ethical clearance was obtained from the Monash University Human Research Ethics Committee (MUHREC Project ID: 47479). Following eligibility screening and an opportunity for potential participants to ask questions, informed consent was obtained. Participants were provided with the consent form electronically and returned a signed copy via email prior to the interview commencing. They were explicitly informed of their right to withdraw at any time before the commencement of data analysis without penalty. All procedures ensured participant confidentiality and anonymity, as detailed throughout this section.

### 3.3. Dataset Generation: Method, Tool, Procedure, and Preparation

Semistructured interviews were selected as the optimal method for dataset generation in this study. The data generation tool was an interview guide developed based on the study’s research questions and informed by the preliminary literature review. It comprised open‐ended questions designed to explore key areas relevant to casual agency nursing, including motivations, experiences, and trade‐offs, impacts on identity and practice; perceptions of the healthcare system and agencies; future career plans; and professional engagement (Table 2).

**TABLE 2:** Core interview questions.

Can you start by telling me a bit about your nursing career path leading up to working exclusively as a casual agency nurse?
What were the main reasons you decided to work *only* as a casual agency nurse, rather than seeking permanent employment?
What do you see as the main advantages and disadvantages (or trade‐offs) of working this way in your experience?
How, if at all, has working exclusively casually affected how you see yourself as a professional nurse—your professional identity?
How do you feel this way of working impacts your connection to colleagues or engagement with the broader nursing profession (e.g., unions, associations)?
From your perspective, what aspects of permanent nursing roles might push nurses towards choosing agency work?
How do you think the healthcare system, specific hospital practices, or agency operations enable or encourage casual nursing careers?
How do you view the long‐term sustainability of working exclusively as a casual agency nurse for your own career? What are your future intentions?
If you could suggest changes to policymakers, hospitals, or agencies regarding casual nursing or permanent roles, what would they be?
Is there anything else you feel is important to share about your experiences as a casual agency nurse that we haven’t discussed today?

The procedure for data generation involved conducting interviews remotely via online video conferencing platforms, selected based on participants’ preferences and accessibility. This remote approach facilitated recruitment across the Melbourne metropolitan area. It offered participants convenience and the ability to select a private, comfortable setting for the interview, with the online space constituting the interview environment. Each individual interview lasted approximately 60–90 minutes, scheduled at a time mutually agreed upon by the participant and the researcher.

Data preparation began with audio‐recording each interview using the secure recording feature of the video conferencing platform. I transcribed each interview verbatim shortly after its completion, allowing for early immersion in the data and ensuring accuracy by meticulously checking transcripts against the audio recordings. Deidentification was performed during transcription: Pseudonyms (RN 1–16) were assigned, and all names of people, specific workplaces (beyond generic descriptions), and locations (aside from Melbourne) were removed to protect confidentiality.

### 3.4. Data Analysis

I conducted an RTA following an iterative, interpretive, and reflexive process [30, 31, 41]. Familiarization involved repeated listening to the audio recordings alongside close reading of transcripts, during which I recorded initial impressions, questions, and tensions in a reflexive journal. Coding then proceeded through two primary coding cycles. In the first cycle, I worked systematically through each transcript line‐by‐line, generating initial, data‐led (primarily inductive) codes that captured both semantic content and more latent meanings (e.g., identity negotiation, resistance narratives, and system critique). Because no qualitative data analysis software was used, I coded directly on transcripts using structured annotation and maintained an evolving code list in a separate analytic document to support transparency and reflexive tracking. In the second cycle, I returned to the transcripts and my initial codes to refine code definitions, collapse overlapping codes, and develop more interpretive “focused” codes that better captured patterns of shared meaning across the dataset. Importantly, this evolving code list was not treated as a fixed “codebook” to be applied consistently for reliability; rather, consistent with RTA, it functioned as a flexible record of my deepening engagement with the dataset and the analytic decisions shaping coding over time.

A worked example of this progression can be seen in the development of Theme 2 (“Opting Out”: Casual Work as Strategic Resistance to Permanent System Failures). In my first coding cycle, I coded RN9’s account of “*escaping the bureaucracy that felt like it was crushing the actual job*” (RN9) with initial labels such as bureaucracy displacing care, nonclinical workload burden, and reclaiming time/focus for “real nursing;” in parallel, RN4’s account of management “*not having our backs*” and feeling “*utterly expendable*” (RN4) was initially coded as lack of organizational support, feeling devalued/disposable, and contract feels ”honest.” In the second coding cycle, these were refined into more interpretive, focused codes that captured patterned meaning across cases (e.g., withdrawal from organizational demands, rejecting exploitative employment cultures, boundary‐setting as self‐preservation, and reframing agency work as career self‐governance). When I collated these focused codes with other related extracts (e.g., accounts of rigid rosters, inability to refuse unsafe shifts, and “the power to say no”), they initially sat under a candidate theme I had tentatively labeled “control and flexibility;” however, through memoing and iterative review against the dataset, I reinterpreted this cluster as doing more than describing preference—it repeatedly positioned agency‐only work as an *active response to perceived system failure* (bureaucracy, burnout culture, and constrained autonomy). This shift in interpretation led me to refine the central organizing concept as resistance‐through‐withdrawal and boundary‐setting, resulting in the final theme title and narrative framing of *“Opting Out:” Casual Work as Strategic Resistance to Permanent System Failures*. An illustrative work example demonstrating the analytic progression from a raw transcript excerpt, through interpretive segmentation and coding refinement to a final theme, is provided in Table 3.

**TABLE 3:** Illustrative progression from meaning units to emerging themes.

Meaning unit	Initial interpretive segment	Initial label	Emerging interpretive grouping	Emerging theme
“You’re there to fill a gap… but you’re not of the place… It’s less personal, more… functional.” (Derived from RN14, Theme 1)	Participant perceives role as external service provider, lacking deep organizational connection.	Transactional relationship	Shifting professional relationships	Theme 1: Crafting a New Professional Self: Identity Beyond the Ward
“I have to compartmentalize… maintain a professional distance… for yourself.” (Derived from RN7, Theme 1)	Conscious emotional boundary‐setting described as necessary for self‐preservation in agency work.	Detachment coping	Managing emotional demands	Theme 1: Crafting a New Professional Self: Identity Beyond the Ward
“My main skill now is fitting in any setting very quickly.” (Derived from RN8, Theme 1)	Professional competence is reframed as rapid adaptation and versatility across sites rather than deep expertise in one context.	Rapid adaptation/versatile generalist	Prioritizing adaptability over deep specialization	Theme 1: Crafting a New Professional Self: Identity Beyond the Ward
“I escaped the bureaucracy that felt like it was crushing the actual job.” (Derived from RN9, Theme 2)	Bureaucracy and nonclinical demands are experienced as displacing “real nursing;” agency work is framed as reclaiming time, focus, and control over how nursing labor is spent.	Bureaucracy displacing care/reclaiming clinical focus	Withdrawal from organizational demands (bureaucracy) as resistance and career self‐governance	Theme 2: “Opting Out:” Casual Work as Strategic Resistance to Permanent System Failures
“They just didn’t have our backs… Management was invisible… I left because I felt utterly expendable.” (Derived from RN4, Theme 2)	Perceived organizational abandonment and managerial invisibility produce feelings of being devalued/disposable; agency work is positioned as a boundary‐setting move toward a more “honest” transactional relationship.	Organizational abandonment/expendability	Rejecting unsupportive/burnout cultures through withdrawal and boundary‐setting	Theme 2: “Opting Out:” Casual Work as Strategic Resistance to Permanent System Failures
“The best part is the power to say ‘no’… If an assignment sounds dodgy… I just don’t take it… It’s like I took my career back into my own hands.” (Derived from RN10, Theme 2)	Ability to refuse unsafe or undesirable shifts is experienced as reclaiming control and autonomy after feeling dictated to in permanent roles.	Power to refuse/selective engagement	Reclaiming professional agency through choice and boundary‐setting	Theme 2: “Opting Out:” Casual Work as Strategic Resistance to Permanent System Failures
“Hospitals use agency… [it’s] flexible for them too… Their own staffing models often make us essential.” (Derived from RN13, Theme 3)	Participant perceives organizational reliance on agency staff for operational flexibility/needs.	Organizational reliance	Systemic enablers of casual work	Theme 3: The Casualization Ecosystem: How Policy and Practice Enable Transient Careers
“System is chronically underfunded… Management says no budget for permanent… but then they pay triple for agency.” (Derived from RN16, Theme 3)	Perceived policy/funding failures create conditions where agency work becomes necessary/attractive.	Policy/funding gaps	Systemic enablers of casual work	Theme 3: The Casualization Ecosystem: How Policy and Practice Enable Transient Careers
“Honestly, I couldn’t manage working across five different hospitals without the agency coordinating it all… They handle the compliance paperwork, the shift booking, chasing payments.” (Derived from RN1, Theme 3)	Agencies are experienced as infrastructure that makes multisite, transient work logistically feasible and sustainable.	Agency as labor‐market intermediary/logistical buffer	Agencies as infrastructure enabling casual “lifestyle”	Theme 3: The Casualization Ecosystem: How Policy and Practice Enable Transient Careers
“It feels like you’re constantly earning just for today… there’s no safety net.” (Derived from RN11, Theme 4)	Anxiety expressed about lack of long‐term financial security (pension, leave) in agency work.	Financial insecurity (future)	Career path uncertainty/precarity	Theme 4: Sustainable Path or Dead End? Career Trajectories and Future Intentions
“You get used to the pay… going back to a staff nurse wage would be really tough now… You worry you might get stuck.” (Derived from RN5, Theme 4)	Difficulty envisioning return to permanent roles due to financial/flexibility adjustment.	Potential “trap”	Barriers to transition/sustainability	Theme 4: Sustainable Path or Dead End? Career Trajectories and Future Intentions
“It works for me now, with young kids, but long term? I honestly don’t know how you’d manage retirement.” (Derived from RN6, Theme 4)	Present‐focused fit (family/life stage) is juxtaposed with uncertainty and anxiety about long‐term viability and retirement.	“Works‐for‐now” framing/future anxiety	Temporal tension between current benefits and perceived future unsustainability	Theme 4: Sustainable Path or Dead End? Career Trajectories and Future Intentions
“It feels… geared towards nurses in permanent… roles. You feel a bit disconnected from that mainstream professional world.” (Derived from RN8, Theme 5)	Sense of isolation from traditional professional structures and associations due to work mode.	Professional disconnection	Fragmented professional engagement	Theme 5: A Fragmented Voice? Casual Work’s Impact on Professional Advocacy & Engagement
“It makes it harder for the unions to argue for better conditions… agency work sort of splinters that voice.” (Derived from RN10, Theme 5)	Concern that workforce fragmentation weakens collective bargaining power and professional advocacy.	Weakened collective voice	Challenges to collective action/advocacy	Theme 5: A Fragmented Voice? Casual Work’s Impact on Professional Advocacy & Engagement
“It’s hard to get involved – shifts are irregular, you don’t know colleagues well enough to organize, and you’re just focused on getting through the day.” (Derived from RN12, Theme 5)	Irregular schedules and weak workplace ties are experienced as practical barriers to union/professional participation and organizing.	Irregular work undermining organizing capacity	Practical obstacles to collective participation	Theme 5: A Fragmented Voice? Casual Work’s Impact on Professional Advocacy & Engagement

## 4. Analysis

**FIGURE 1:**
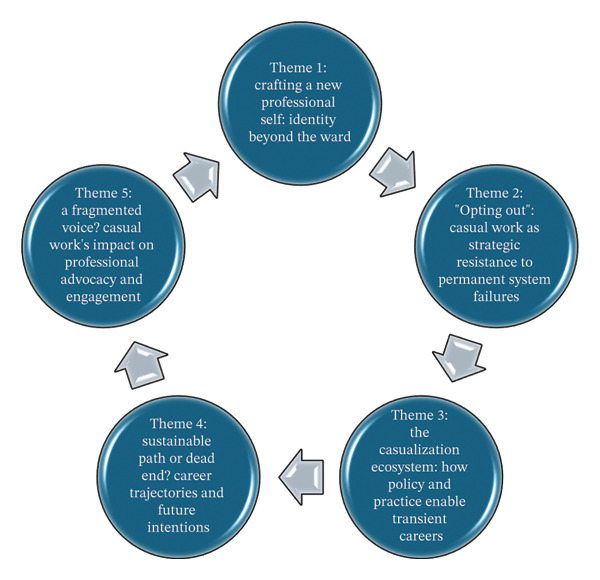
Thematic map of core themes: Identity, resistance, systemic factors, career paths, and professional engagement in casual agency nursing.

The analysis of interviews with 16 casual agency nurses revealed profound shifts in their experience and conceptualization of professional nursing identity. Figure 1 depicts a set of interconnected, coexisting interpretive domains representing the themes generated during the data analysis process.

### 4.1. Theme 1: Crafting a New Professional Self: Identity Beyond the Ward

Sustained engagement in casual agency nursing appears to catalyze a reconstruction of professional identity, moving nurses from organizationally embedded roles toward defining themselves as more independent, adaptable, yet sometimes detached practitioners. This theme unpacks that identity shift, revealing a complex interplay between the structural demands of agency work and nurses’ active negotiation of their professional self, echoing identity theories that emphasize contextual, fluid, and relational understandings of nursing professionalism [50–52]. Key facets of this reconstructed identity include a more transactional orientation to work relationships, the strategic use of emotional distance, and a reprioritization of professional skills toward adaptability, particularly among participants with several years of agency‐only experience.

Participants frequently described their work relationships—with both agencies and healthcare facilities—in functional, transactional terms, diverging from narratives of belonging and mutual obligation often associated with permanent roles [53]. The nature of short‐term assignments positioned them as external resources that fulfill a specific need, fostering an identity akin to that of an independent contractor or “free agent” prevalent in contingent work studies [54, 55]. This pragmatic stance focused on clear deliverables and contractual obligations, as RN5 noted, “You go in, do the job, get paid, leave. It’s clean.” RN3, who had several years of agency‐only work, similarly reflected, “I see myself more as a professional service than part of a hospital ‘family.’ I bring a skillset for the shift, and that’s the agreement” (RN3). This functional identity was further elaborated by RN14, who highlighted the feeling of operating outside the organizational “family:”

You definitely feel like an outsider, always. You’re not invited to the unit meetings, you don’t get the internal emails, you don’t really know the long‐term patients or the little quirks of the ward politics. You’re there to fill a gap, do your tasks competently, and be professional, but you’re not “*of”* the place. It’s a service you provide, like a consultant coming in. There’s a distance […] It’s less personal, more functional. (RN14)

This *transactionalism*, potentially reflecting broader neoliberal trends that marketize healthcare work, not only offers autonomy but also challenges professional models emphasizing collegiality, embeddedness, and symmetrical psychological contracts between nurses and organizations [56–58]. For nursing management, it raises questions about how to maintain team cohesion, shared values, and a sense of professional community when a sizeable segment of the workforce experiences themselves as mobile, contract‐based providers rather than long‐term organizational members [59, 60].

Alongside this transactional stance, many nurses described actively cultivating *emotional detachment* as a necessary strategy for navigating the demands of agency work. The constant movement between unfamiliar settings and transient patient encounters made deep relational investment difficult and potentially draining. This practice, distinct from mere indifference, was framed as crucial for self‐preservation and preventing burnout, aligning with work on emotional labor and emotional distancing as a protective, professionalism‐preserving coping strategy in high‐stress care environments [61, 62]. “You have to have thicker skin,” RN11 stated, “You can’t get too attached […] It helps you cope.” RN7 described the conscious effort required:

It′s a conscious approach. In my old permanent job, I knew all my patients, their families […] Now? I focus on safe, efficient care for the shift I am on. I have to compartmentalize. If I invested the same emotional energy everywhere I went, I′d burn out in a month […] You maintain a professional distance, not just for them, but for yourself. (RN7)

Similarly, RN2 reflected, “If I connected as deeply in every new ICU as I did in my ‘home’ unit, I wouldn’t last 6 months. So, you learn to draw a line in a different place” (RN2). While potentially adaptive, this cultivated detachment creates tension with nursing’s relational ethos and historical notions of *“detached concern”* [63, 64]. It prompts consideration of how nurse managers and organizations can support emotionally sustainable practice in models of work that structurally limit continuity and depth of relationships, without eroding the caring core of nursing.

Finally, these reconstructed professional self‐involved re‐evaluating skills prioritize adaptability over specialization. Participants consistently highlighted their capacity to quickly orient, apply diverse procedural skills, and function effectively in new environments as core competencies developed through agency work. RN8 noted, “My main skill now is fitting in any setting very quickly.” RN1, early in her agency career, similarly observed, “I feel like I’ve become an all‐rounder who can handle most wards and that’s what I’m proud of” (RN1). This emphasis aligns with boundaryless and protean career concepts and with the need for functional flexibility in managing healthcare staffing fluctuations [65, 66]. However, this often occurred alongside an acknowledged trade‐off regarding opportunities for specialized expertise, as RN12 reflected:

You become really good at walking onto a ward […]. However, do I feel like an expert in cardiac nursing as I used to ? No. You lose that deep dive […]. Your expertise becomes about being a competent generalist who can handle anything and everything, rather than specializing in a particular area. (RN12)

This focus on adaptability challenges traditional nursing career trajectories and frameworks for specialization and continuing professional development, which remain largely designed around stable, organizationally anchored roles [67, 68]. It suggests a need for workforce and management policies that recognize and scaffold the specific skill‐set of agency nurses—such as rapid role adaptation and cross‐contextual competence—while ensuring access to structured development pathways and mentorship so that “generalist‐flexible” careers do not become structurally second class.

Taken together, these facets—transactionalism, strategic detachment, and an adaptability‐centered skill profile—create a professional identity that is qualitatively different from traditional models based on organizational loyalty, linear specialization, and relational continuity. This evolving identity “beyond the ward” presents fundamental challenges to established concepts of nursing professionalism and to nurse management practices premised on stable teams. It underscores the importance of leadership approaches and job design that can accommodate, integrate, and support agency nurses as legitimate members of the professional community, even when their contractual and identity orientations are less organizationally anchored.

### 4.2. Theme 2: “Opting Out:” Casual Work as Strategic Resistance to Permanent System Failures

Beyond constructing a new professional self, the decision to work exclusively via agencies was frequently framed by participants not merely as a passive preference for flexibility, but as an active, strategic “opting out”—a form of resistance against perceived systemic failures within traditional, permanent healthcare employment structures. Nurses described casual work as a way of responding to what they experienced as chronic breaches of the implicit psychological contract between themselves and their employers—unmet expectations around support, fair workload, and recognition—echoing scholarship that links psychological contract breach to turnover intentions and even exit from the occupation [69–72].

Participants consistently described escaping the *inflexibility* and *bureaucratic burdens* endemic to many permanent positions as a primary motivator. Rigid rosters unresponsive to personal needs, mandatory non‐clinical tasks, “pointless meetings,” and hierarchical communication channels were cited as significant frustrations. Casual agency work, in contrast, offered a release from these constraints, providing direct control over scheduling and circumventing much of the administrative overhead. As RN2 stated, “I just couldn′t deal with the rosters anymore […] being told when I had to work years in advance […], no negotiation. Agency work means I decide.” This desire for autonomy resonates with research demonstrating that work–life balance and scheduling control are central to nurse retention and intention to stay [73–76]. One nurse vividly contrasted the perceived freedom of agency work with the constraints of her previous permanent role:

The amount of time wasted in permanent jobs on things that weren′t patient care was staggering. Unit meetings, mandatory training on things irrelevant to my specific skills, endless emails, the politics […] Here [agency work], I get the call, I check the details, I go, I nurse, I leave. It’s pure nursing, in a way. I escaped the bureaucracy that felt like it was crushing the actual job. It was about reclaiming my time and my focus. (RN9)

Furthermore, participants frequently narrated their shift to agency work as a reaction against unsupportive work cultures and the pervasive risk of burnout in permanent roles. These accounts parallel broader evidence linking unsupportive management, poor organizational culture, and lack of recognition to burnout and retention problems in nursing [77–79]. Agency work, despite its own insecurities, was often positioned as a healthier alternative, allowing nurses to set boundaries and avoid environments they deemed toxic or detrimental to their mental health [80–82]. “My old hospital just burned people out and expected them to keep going,” RN15 commented, “Agency lets me step away before I hit that wall.” The sentiment of employers failing to support their staff was a powerful, recurring element:

They just didn’t have our backs during the worst of it [post‐COVID pressures]. Management was invisible, resources were stretched, and they just expected more and more. There was no recognition, no real support. With the agency, at least it feels honest – it’s a contract for a shift. They don’t pretend to be family, but they also don’t demand your soul. I left my permanent job because I felt utterly expendable; bizarrely, I feel more respected, or at least less exploited, now. (RN4)

Opting for casual agency work was framed by many as a way to *reclaim* professional agency and exert control over their working lives in a manner impossible within the constraints of their former permanent positions. This extended beyond mere scheduling flexibility to include the ability to choose specific types of facilities or wards, decline assignments in environments perceived as unsafe or poorly managed, and dictate the overall volume of work undertaken. This exercise of choice countered feelings of powerlessness often described in relation to large healthcare organizations [83, 84]. Prior research highlights professional autonomy as a cornerstone of professional satisfaction and identity, and agency work provided a direct mechanism for participants to enact this autonomy [85–87]. As RN10 expressed:

The best part is the power to say ‘no’. If an assignment sounds dodgy, or I know that hospital has terrible staffing, I just don’t take it. You can’t do that in a permanent job; you’re stuck. Here, I’m in the driver’s seat. I choose where I lend my skills. If I want a break, I take one. That control, after years of feeling dictated to, is incredibly valuable. It’s like I took my career back into my own hands. (RN10)

Viewing the move to casual agency work through this lens of resistance suggests that its rise is not simply about market forces or individual desires for flexibility alone. It is also a critique of, and a strategy to cope with, perceived failures within traditional healthcare employment models—inflexibility, demanding and unsupportive cultures, and constraints on professional autonomy [88–90]. For nurse managers and organizational leaders, this theme underscores that efforts aimed solely at incentivizing permanence (e.g., financial bonuses) are unlikely to be sufficient without addressing the underlying systemic issues—rostering rigidity, workload, and perceived lack of support—that prompt nurses to “opt out” in the first place. Addressing these root causes of resistance within permanent roles is likely crucial for sustainable workforce planning and for rebuilding trust, reciprocity, and a viable psychological contract between nurses and their employing organizations.

### 4.3. Theme 3: The Casualization Ecosystem: How Policy and Practice Enable Transient Careers

While nurses actively craft new identities and strategically opt out of permanent roles, their ability to sustain exclusively casual‐agency careers is deeply embedded in a broader *“casualization ecosystem”* [26, 91]. This theme illuminates how participants perceived specific health policies, organizational practices, and the functioning of the agency market itself as creating and maintaining the conditions under which transient nursing careers flourish. Moving beyond individual motivations, the analysis highlights systemic enablers of casualization, resonating with work on the casualization of the nursing workforce in Australia and elsewhere, and with systems‐thinking accounts of how local staffing practices interact with wider funding and reform agendas [65, 92, 93]. Participants identified organizational reliance on flexibility, the mediating role of agencies, and perceived policy gaps as key components of this ecosystem.

Participants frequently pointed to the perceived operational benefits and strategic reliance of healthcare organizations on agency staff. They described facilities using casual nurses not only for emergencies but also routinely to manage fluctuating patient census, cover chronic short‐staffing on permanent rosters, fill specialist vacancies, or potentially mitigate the costs associated with permanent employee entitlements [94, 95]. This suggests a normalization of contingent labor within hospital staffing models. As RN6 observed, agency nurses are often needed “because the wards are constantly short.” This reliance creates a continuous demand that sustains the viability of choosing exclusive casual work. One nurse offered a pragmatic perspective on why facilities depend on agency staff.

Look, from the hospital’s point of view, I get it. They have budget lines, and they have peaks and troughs in patient load. Bringing in an agency means they only pay for the hours they absolutely need, without the ongoing costs—the sick leave, the annual leave, the super contributions (regular payment made into a fund by an employer toward a future pension), and sometimes the professional development budgets. It is flexible for them too. We (agency nurses) are an easily adjustable workforce volume. While they complain about agency costs, their own staffing models often make us essential. (RN13)

Participants’ accounts here resonate with analyses showing that, in the short term, organizations may view temporary staff as a flexible “safety valve,” even though over‐reliance on agency labor can inflate operating costs and complicate efforts to achieve safe, stable staffing [96–98]. From a nursing management perspective, this theme underscores the importance of aligning staffing strategies and budgetary frameworks with longer‐term workforce sustainability, rather than treating agency labor as an endlessly elastic buffer for structural understaffing.

Central to this ecosystem is the enabling role of the nursing agencies themselves. Participants described agencies as crucial intermediaries that streamline access to work, manage administrative burdens (such as credentialing, timesheets, and payments), and sometimes act as advocates or buffers between the nurse and the facility. “The agency is like your agent,” RN3 commented, “they find the gigs, you just turn up.” This convenience factor was significant, allowing nurses to focus on clinical work rather than job searching or complex negotiations with multiple employers. Agencies effectively create and manage the marketplace, connecting flexible labor supply with fluctuating organizational demand [99, 100]. RN1 reflected on the practical benefits:

Honestly, I couldn’t manage working across five different hospitals without the agency coordinating it all. They handle the compliance paperwork, the shift booking, chasing payments […] It makes being ‘independent’ actually feasible. Yes, they take a cut; everyone knows that, but what service do they provide to find consistent work and simplify the logistics? For me, right now, it is worth it. They make the whole ‘casual lifestyle’ possible on a practical level. (RN1)

However, this reliance also positions agencies as powerful actors within the ecosystem, profiting from workforce transience and potentially shaping market conditions. Their role highlights the need for policy considerations regarding agency regulation, fee transparency, and fair labor practices within this mediated employment relationship.

Finally, participants often pointed toward perceived policy gaps or the unintended consequences of broader health system settings as contributing to the casualization ecosystem. This included critiques of inadequate government funding, which led to persistent understaffing in public hospitals, making permanent roles highly stressful and unattractive [101]. Others mentioned aspects of industrial agreements or enterprise bargaining agreements (EBAs) that, while designed to protect workers, were sometimes seen as creating rigidities that organizations circumvented through agency use [102, 103]. There was a sense that policy often failed to adequately address the root causes of workforce dissatisfaction or regulate the burgeoning flexible labor market effectively [104, 105]. Participants’ accounts resonate with wider critiques of neoliberal reforms, which suggest that policies prioritizing cost efficiency, market mechanisms, and “lean” staffing can inadvertently promote workforce fragmentation and precarious employment, with implications for worker well‐being and care quality [106]. One nurse linked their choice directly to systemic funding issues:

The public system is chronically underfunded. Wards run short every single day. So you have permanent staff who are exhausted and burnt out, taking on all the extra load. Management says there′s no budget for more permanent hires, but then they pay triple for agency to plug the gaps. It makes no sense financially long‐term, but that′s the policy reality. So why would I stay in a permanent role to be overworked and under‐supported when the system itself creates this huge demand for better‐paid, flexible agency work? It feels like the policies push you out. (RN16)

This perspective underscores how macro‐level policy decisions regarding funding, industrial relations, and workforce planning create the environment within which both nurses and healthcare organizations make their choices. Critiques aligned with analyses of neoliberal reforms suggest that policies prioritizing efficiency or market mechanisms may inadvertently promote workforce fragmentation and precarity [107, 108]. Addressing the casualization trend, therefore, requires moving beyond individual or organizational levels to examine and potentially reform the broader policy and funding landscape that constitutes this enabling ecosystem.

### 4.4. Theme 4: Sustainable Path or Dead End? Career Trajectories and Future Intentions

While participants articulated clear rationales for choosing and navigating casual agency work in the present, their perspectives on the long‐term sustainability and future trajectory of this career path were marked by considerable tension and uncertainty. This theme explores the dichotomy participants experienced when contemplating their future in nursing: viewing exclusive agency work as either a potentially sustainable, albeit demanding, path requiring careful management, or as a temporary phase that could lead to a career dead end or exit from the profession. This uncertainty contrasts sharply with traditional, organizationally structured nursing careers that often feature clearer progression pathways [109] and highlights the challenges of long‐term planning within the precarious conditions often associated with the gig economy or contingent work [110].

A dominant narrative involved a pronounced sense of *ambiguity* about the future, often expressed as an “it works for now” mentality. Many participants, while satisfied with the current benefits of flexibility and income, voiced significant concerns about the long‐term viability of relying solely on agency work. This anxiety stemmed from the inherent unpredictability of income, the lack of employer‐sponsored retirement plans or benefits such as long service leave common in Australian permanent roles, concerns about ageism affecting shift availability later in their careers, and the potential physical toll of constantly adapting to new environments [111–113]. As RN6 reflected, “It works for me now, with young kids, but long term? I honestly don’t know how you’d manage retirement.” This resonates with broader studies highlighting the financial insecurity and lack of safety nets for gig workers [114, 115]. One nurse expressed a deep‐seated worry about the lack of a future buffer:

The money is good now, definitely better per hour than my old permanent job. But there’s nothing building up for the future. No proper superannuation, no sick leave buffer if I get seriously ill, no long service leave down the track. It feels like you’re earning just for today. If the work dried up, or if I couldn’t physically do these demanding shifts anymore […] there’s no safety net. That’s the part that keeps you awake sometimes, wondering if you need an exit strategy. (RN11)

For some, this uncertainty morphed into a feeling that exclusive agency work could become a potential “trap.” Having adapted to the higher hourly rates and autonomy, participants described it as financially or psychologically difficult to consider returning to the perceived constraints and lower base pay of permanent positions, even if they desired greater stability. This sense of being caught—enjoying the present benefits while lacking a clear path forward—created inertia. “You get used to the agency [casual rate] pay,” RN2 admitted, “going back to a [permanent] registered nurse wage would be really tough now, even with the benefits.” This suggests a form of path dependency where the immediate advantages of casual work make transitioning back to traditional structures challenging. Another participant described this feeling of being caught:

It’s like golden handcuffs sometimes. The flexibility is addictive, and the pay makes it hard to justify a permanent role unless it’s perfect. But this probably isn’t forever. You′re not really progressing in a traditional sense, maybe not building specific skills for a higher role. So you keep doing it because it works now, but you don′t see a clear ‘next step’ within this model, and going back feels like a step down financially. You worry you might get stuck. (RN5)

However, the narrative was not uniformly uncertain. A subset of participants viewed exclusive agency work as a potentially sustainable career path, achieved through proactive strategies. These nurses emphasized meticulous financial planning, such as independent superannuation contributions, budgeting for downtime, continuously updating skills across diverse areas to remain marketable, cultivating strong relationships with agencies, and sometimes combining agency work with other entrepreneurial activities. This suggests that for some, agency nursing can be navigated long‐term but requires a high degree of individual responsibility and planning, often supported structurally within permanent roles. Contrasting this were participants who explicitly viewed agency work as temporary or transitional [116], using it to fund further education for a different career path, gain specific experience before seeking a particular permanent role, or as a way to phase out of nursing altogether, reflecting broader trends of nurse turnover. One nurse planning an exit explained:

This agency gig is a means to an end for me. I’m saving aggressively to go back and do my Master’s in health administration. I enjoy the clinical work, but I don’t see a sustainable future for myself doing only this—the instability, the lack of career progression. It’s been great for flexibility while figuring things out and earning good money, but it was never the long‐term plan. Another couple of years, then I’m out. (RN16)

Ultimately, this theme reveals significant heterogeneity in future intentions but underscores pervasive uncertainty about the sustainability of exclusive casual agency careers. Whether viewed as a manageable path, a temporary phase, or a potential trap, these trajectories carry weight for workforce planning and policy. If a substantial number of experienced nurses perceive this work model as ultimately unsustainable or primarily as an exit route, it exacerbates workforce churn and the loss of embedded organizational knowledge. Policy considerations must therefore extend beyond managing current staffing needs to fostering career structures—both within traditional and nontraditional employment models—that offer genuine long‐term sustainability and security for the nursing workforce.

### 4.5. Theme 5: A Fragmented Voice? Casual Work’s Impact on Professional Advocacy and Engagement

The preceding themes illustrate how exclusive casual agency work reshapes individual nurses’ identities, motivations, and career perspectives. This final theme examines the collective dimension, exploring how this mode of employment affects participants’ connection to the broader nursing profession, their engagement with representative bodies such as unions and professional associations, and their perceived collective influence on policy and practice. The analysis suggests that the structural conditions of casual agency work often contribute to a fragmented professional voice, posing challenges for collective identity, representation, and advocacy. This resonates with broader scholarship showing that professional associations and unions are critical vehicles for health‐worker policy influence [117, 118], yet their traditional models of membership and participation are strained by nonstandard and platform‐like work arrangements that disperse workers across sites and employers [118, 119].

Participants frequently described a sense of disconnection from the day‐to‐day collegial and professional activities centered around specific healthcare facilities or stable teams. Constantly moving between workplaces, limited opportunities for deep professional bonding, and participation in unit‐based activities, including union meetings or quality improvement initiatives, were some barriers identified by the participants. “You miss the camaraderie sometimes,” RN3 admitted, “and you’re definitely out of the loop on ward‐level union stuff because you might not be back there next week.” RN2 similarly reflected, “You’re always the extra pair of hands, never part of the core team, so you don’t really get asked about changes or ward issues” (RN2). This perceived isolation could extend to engagement with larger professional associations, which were sometimes seen as more oriented toward nurses in traditional roles or academic pathways [120, 121]. One nurse described this feeling of being on the periphery:

I’m a member of the [Professional Association], but I rarely go to events or engage much beyond reading the journal. It feels […] geared towards nurses in permanent leadership or education roles. The issues they focus on, like climbing the ladder in one hospital, don’t always resonate with my day‐to‐day reality. You feel a bit disconnected from that mainstream professional world, even though you’re doing the same core nursing work. (RN8)

This disconnection often translated into ambivalence or perceived barriers regarding collective action and representation. While some participants maintained union membership, others questioned its relevance or effectiveness within the agency context, seeing their primary relationship as being with the agency rather than a specific hospital employer amenable to traditional collective bargaining. Others expressed a desire for representation but faced practical hurdles. “It’s hard to get involved,” stated RN12, “shifts are irregular, you don’t know colleagues well enough to organize, and honestly, you’re often just focused on getting through the day.” This reflects challenges noted in the literature regarding the mobilization of contingent and gig workers, where fragmented employment relationships and variable schedules can undermine traditional organizing and collective voice mechanisms [122, 123]. Another participant elaborated on the perceived mismatch between their work style and collective structures:

The union feels like it’s set up for the permanent staff, dealing with the EBA [Enterprise Bargaining Agreement] for that hospital. As an agency nurse, my pay and conditions are set through the agency contract, which feels more like an individual negotiation, even if it’s a standard rate. It just feels like a different system, and the collective approach doesn’t seem to fit easily. (RN6)

This perceived difficulty in collective engagement led some participants to be concerned about a weakening of the overall professional voice and influence. There was concern that a growing, fragmented casual workforce could inadvertently undermine efforts to improve conditions for all nurses by making unified advocacy more challenging, corroborated by the International Council of Nurses (ICN) [124]. Some felt that the individual focus required by agency work, or the perceived division between “loyal” permanent staff and “mercenary” casuals (a stereotype they contested), could hinder collective bargaining power and the profession’s ability to lobby effectively on issues such as safe staffing or scope of practice. One nurse expressed this concern explicitly:

My worry is that if too many experienced nurses go agency, it fragments the workforce. It makes it harder for the unions to argue for better conditions across the board, because a chunk of the workforce operates outside that main agreement. It might even make hospitals less likely to create permanent jobs, because they know they can plug gaps with agency. We need a strong collective voice, but agency work sort of splinters that voice, doesn’t it? (RN10)

Nonetheless, this theme suggests that while casual agency work offers individual benefits, it concurrently presents challenges to collective professional identity, engagement, and advocacy. The fragmented nature of the work can lead to disconnection, create barriers to participation in traditional representative structures, and potentially dilute the collective influence of the nursing profession. Given evidence that nurses’ engagement and sense of professional community are linked to satisfaction, retention, and perceived quality of care [125, 126], these dynamics have direct implications for nursing management and workforce sustainability. The findings highlight a need for professional associations, unions, and nurse leaders to develop innovative strategies—such as dedicated networks for agency nurses, flexible modes of participation, and advocacy structures that recognize triadic employment relationships—to engage, represent, and mobilize this growing segment of the workforce effectively [117, 118]. Furthermore, it underscores the importance of fostering solidarity and addressing potential divisions between permanent and casual staff to maintain a strong, unified professional voice capable of advocating for improvements that benefit all nurses and improve the quality of patient care.

## 5. Final Considerations

This concluding section synthesizes the analytic narrative emerging from the study, articulating the research’s contributions, “limitations” which is reframed as situatedness, and implications for policy and practice. It discusses directions for future inquiry and reflects on the research quality and reflexive process, adhering to the principles of methodologically coherent qualitative reporting outlined in the RTARG guideline.

### 5.1. Situated Knowledge and Transferability

This study produced situated knowledge that is inseparable from the context in which it was generated and interpreted. The analysis reflects the accounts of 16 registered nurses working exclusively via two national nursing agencies with substantial operations in Melbourne, within the specific healthcare, policy, and Victorian industrial relations environment shaping contemporary nursing work in Australia. The dataset was generated through remote interviews, and—as the sole researcher with an acknowledged insider positioning, my interpretations inevitably contribute to shaping the knowledge constructed herein. These factors—the specific location, participant characteristics, timing, and researcher positionality—are part of how my interpretive engagement contributed to the analytic account produced. These features (setting, participant group characteristics, mode of dataset generation, timing amid post‐COVID workforce pressures, and researcher positionality) do not function as “limitations” in a positivist sense; instead, they define the conditions under which this interpretive account was constructed. Accordingly, the study does not claim statistical generalizability. Instead, its potential value lies in analytic transferability: the themes concerning identity reconstruction, strategic withdrawal/resistance, systemic enablement of casualization, career (un)sustainability, and professional voice may offer points of comparison for stakeholders examining nonstandard nursing work in other settings. Such comparisons should be made cautiously and with explicit attention to contextual differences—particularly in regulatory arrangements, employment protections, benefit portability, pay‐setting mechanisms, and labor‐market dynamics—that distinguish Australia’s agency model from, for example, US travel nursing or UK bank staffing.

### 5.2. Final Reflexive Considerations

Several moments during analysis explicitly challenged my assumptions and led to shifts in interpretation. For example, I began the study expecting flexibility and income to be the dominant explanatory frame for agency work; early coding reflected this, with a provisional theme labeled “control and flexibility.” As I revisited the data, however, repeated, emotionally charged accounts of leaving due to burnout, perceived lack of organizational support, and disillusionment with bureaucracy prompted me to re‐read these narratives as forms of critique and resistance, rather than simply as preference. This reflexive reconsideration, aided by discussion with my critical friend, contributed directly to reframing Theme 2 from a focus on flexibility to “opting out” as a form of strategic resistance. Similarly, I initially anticipated a relatively uniform disengagement from professional advocacy among agency nurses; however, closer engagement with accounts from participants who maintained union membership or expressed concern about the collective implications of casualization required me to complicate this assumption and to craft Theme 5 around *fragmented*, rather than absent, professional voice. In addition, some participants carefully planned long‐term agency careers challenged my early tendency to read agency work primarily as temporary or transitional, prompting a more balanced portrayal of Theme 4 that holds together both perceived “trap” dynamics and deliberate, sustainable strategies.

Finally, my insider perspective intersected with the demographic shape of the participant group. The difficulty in recruiting male participants and the resulting predominance of women did not simply function as a limitation to be listed; it also reminded me that my own experience of nursing—shaped by gender, migration, and career stage—represents one situated lens among many. Reflexive engagement required me to remain alert to whose voices were more or less present in the dataset, and to avoid universalizing the experiences of this particular group of Melbourne‐based agency nurses as representative of all agency nurses, even when their accounts resonated strongly with my own. Acknowledging these dynamics and how my assumptions were both affirmed and unsettled by the data is central to the transparency and trustworthiness of the knowledge produced in this study.

### 5.3. Directions for Future Research

Stemming directly from this study’s findings and its situated nature, several avenues for future research warrant exploration to build upon this work. Given the challenges in recruiting male participants, research focusing specifically on the experiences and identity constructions of male nurses engaged in casual agency roles would be particularly valuable. To explicitly examine transferability, multisite studies across different Australian states and territories—and across public, private, and not‐for‐profit providers—could compare how varying industrial agreements, funding models, and agency markets shape agency nurses’ identities, resistance narratives, and career intentions. Cross‐jurisdictional comparative work, for example, contrasting Australia’s regulated agency model with US travel nursing or UK bank staff arrangements, would further test how different policy and regulatory environments condition similar or divergent experiences. Future research should also include manager‐ and agency‐operator–focused studies (e.g., qualitative interviews or case studies of workforce planning and staffing strategies) to provide a systemic counterpoint to the nurse‐focused lens adopted here, and patient‐ and family‐focused studies exploring perceived care continuity, safety, and relational quality in settings with high agency utilization. Finally, quantitative or mixed‐method research could examine the impact of sustained casualization on the development and maintenance of specialized nursing skills within the broader workforce—for example, linking workforce datasets with in‐depth qualitative accounts—to assess how agency‐dominated career patterns influence skill mix, leadership pipelines, and organizational knowledge over time.

### 5.4. Analytic Conclusions and Actionable Implications

The findings indicate that exclusive agency nursing is not a marginal or purely instrumental employment choice, but a configuration of practice that fundamentally challenges conventional models of nursing work, career development, and professional engagement.

The central contribution of this research is to demonstrate that casual agency nursing should be understood as both an individual adaptive strategy and a systemic signal. Simply enhancing financial incentives for permanent positions is unlikely to be effective without addressing the underlying conditions that participants identified as catalyzing their “opt out:” inflexible and opaque rostering systems, entrenched burnout cultures, and perceived deficits in managerial support and recognition [127–129]. Concurrently, the ecosystem that enables casual work warrants examination; organizational reliance on transient labor and the mediating role of agencies require scrutiny [93, 130]. Therefore, actionable implications span multiple levels.

• For health service management: There is a need to systematically review and redesign permanent role structures to incorporate meaningful flexibility, predictable and participatory rostering, and visibly supportive leadership, thereby addressing key drivers of resistance and exit. Practical strategies may include piloting self‐scheduling or team‐based rostering models, embedding structured debriefing and well‐being supports, and developing clear orientation and communication protocols to integrate agency staff into ward teams, ensuring care quality, accountability, and cohesion are maintained.
• For policy makers and system stewards: Workforce‐relevant policies, including funding models and staffing benchmarks, should be reviewed for unintended incentives that promote casualization over the maintenance of robust permanent establishments. Regulatory frameworks for nursing agencies merit attention regarding transparency in fee structures, minimum employment standards, and quality assurance processes. Industrial relations’ arrangements may need to be adapted to more fully encompass nontraditional work models, ensuring that nurses working through agencies have access to equitable conditions, protections, and professional development opportunities.
• For professional bodies and unions: Targeted strategies are required to engage, represent, and support the growing cohort of nurses working outside traditional organizational employment. This may entail developing dedicated membership categories or networks for agency nurses, offering flexible modes of participation (e.g., online forums and virtual assemblies), and explicitly incorporating agency perspectives into advocacy agendas. Fostering solidarity and reducing perceived divisions between permanent and casual staff is essential for sustaining a unified professional voice capable of influencing systemic issues such as safe staffing, fair remuneration, and the future design of nursing careers.

## Author Contributions

Conceptualization: Animesh Ghimire; methodology: Animesh Ghimire; validation: Animesh Ghimire; formal analysis: Animesh Ghimire.

Investigation: Animesh Ghimire; data analysis: Animesh Ghimire; manuscript writing–original: Animesh Ghimire; manuscript writing–review and editing: Animesh Ghimire.

## Funding

Open access publishing facilitated by Monash University, as part of the Wiley ‐ Monash University agreement via the Council of Australasian University Librarians.

## Disclosure

The author has read and approved the submitted version of the manuscript.

## Ethics Statement

This study was conducted in accordance with the ethical principles outlined in the Declaration of Helsinki. Ethical clearance was obtained from the Monash University Human Research Ethics Committee (MUHREC Project ID: 47479).

## Consent

All participants consented to publication. All participants consented verbally and with a written consent form.

## Conflicts of Interest

The author declares no conflicts of interest.

## Supporting Information

The following Supporting Information is available online to accompany this article: Supporting File 1: Reflexive Thematic Analysis Reporting Guidelines (RTARG). This document provides the full Reflexive Thematic Analysis Reporting Guidelines (RTARG) by Braun and Clarke, which informed the reporting of this research and offers detailed guidance on ensuring methodological coherence and reflexive openness.

## Supporting information


**Supporting Information** Additional supporting information can be found online in the Supporting Information section.

## Data Availability

The data that support the findings of this study are available on request from the corresponding author. The data are not publicly available due to privacy or ethical restrictions.
